# Understanding Ricin from a Defensive Viewpoint

**DOI:** 10.3390/toxins3111373

**Published:** 2011-11-04

**Authors:** Gareth D. Griffiths

**Keywords:** toxicology of ricin, inhalation, parenteral (intramuscular), ingestion, detection, antibody based tests, mass spectrometry, polymerase chain reaction

## Abstract

The toxin ricin has long been understood to have potential for criminal activity and there has been concern that it might be used as a mass-scale weapon on a military basis for at least two decades. Currently, the focus has extended to encompass terrorist activities using ricin to disrupt every day activities on a smaller scale. Whichever scenario is considered, there are features in common which need to be understood; these include the knowledge of the toxicity from ricin poisoning by the likely routes, methods for the detection of ricin in relevant materials and approaches to making an early diagnosis of ricin poisoning, in order to take therapeutic steps to mitigate the toxicity. This article will review the current situation regarding each of these stages in our collective understanding of ricin and how to defend against its use by an aggressor.

## 1. Introduction

The protein toxin, ricin, is effectively a bi-product of the castor oil extraction process and at least 100 million metric tonnes of castor beans are grown and processed every year with the purpose of preparing castor oil. In addition to cultivation for oil production, the *Ricinus* plant also grows like a weed in warmer climates of the world, including Africa, the Middle East, India and Southern France. A highly toxic preparation may fairly easily be produced from castor bean seeds and recipes have been circulated on the internet [1,2,3,4] and in manuals that would assist the would-be terrorist. The terrorist “cookbooks” have been examined by Pita and Domingo [5] and, in the case of ricin, deemed incapable of achieving a good product for causing a large number of casualties by any exposure route, mainly because of the low content of toxin of the final extracts. It is a large step from preparing a poisonous mixture from castor beans, to delivering this material in a suitable form, on a large enough scale, to cause harm to a number of target subjects. Ricin is harmful and can have potentially lethal consequences when delivered by several routes, including those which are considered relevant to military or terrorist scenarios, namely inhalation, parenteral administration (e.g., transdermal, sub-cutaneous or intramuscular) or by ingestion. However, the toxicity of the toxin may be very different depending upon the route of intoxication. Franz and Jaax [6] have presented a collection of toxicity assessments for ricin pertaining to different routes of exposure based on studies in mice. The toxicity of ricin in mice causing death in 50% of the target population (LD_50_) by inhalation is given as 3-5 μg kg^−1^ with a mean time to death of 60 h; 24 μg kg^−1^ and death in 100 h by subcutaneous injection and 20 mg kg^−1^ and death in 85 h by ingestion. The main lesion caused by ricin is impairment of the protein synthesis machinery of the cell [7] but, in the living organism, a number of symptoms arise leading to an overall picture of ricin intoxication. In the following section, the toxicity, signs and symptoms of ricin poisoning will be examined, as far as is possible, by route of intoxication. 

If ricin were released in an attack, it is essential that the toxin is detected on materials and this might entail extremes such as sampling soil to foodstuffs, particularly if contamination of foods had been threatened. As a result of this risk, a considerable amount of work has been undertaken over the past few years to develop detection methods for ricin in a range of substrates. The detection of ricin will be examined, giving a flavour of methods attempted and limits of detection achieved. 

Finally, the ability to diagnose exposure to ricin toxin is an important prelude to subsequent therapy, time being of the essence. Attempts currently underway that inform the diagnosis of ricin poisoning will be examined.

## 2. Inhalation of Ricin

Using a Liu-Lee aerosol generator [8], which produces a tight mass median aerodynamic diameter (MMAD) aerosol particle of just below 1 μm, Porton Wistar rats, (mean bodyweight 220 g) were exposed to aerosols generated from solutions of two different preparations of pure ricin. Ricin prepared from the Hale Queen variety of the castor oil plant was obtained commercially (Sigma) while the second was prepared in house from seeds of *Ricinus communis* var. *zanzibariensis*. Aerosol exposure times and solution concentrations were varied to achieve a range of exposure doses (the product of aerosol concentration, C, and time, t; Ct has units of mg min m^−3^). It is conventional in inhalation toxicology to express (lethal) challenges as the product of C and t, rather than as lethal dose (LD). This is because the actual dose retained in the lungs is often uncertain and many studies do not attempt to calculate the actual retained fraction of the inhaled aerosol. The details of sample preparations, exposure conditions and subsequent inhalation toxicity data have been published in full [9]. The LCt_50_ values determined for the toxicity of the two preparations were different, being between 4.54 and 5.96 mg min m^−3^ (estimated LD_50_ 3.7 μg kg^−1^) for Hale Queen and 12.7 mg min m^−3^ (estimated LD_50_ 9.8 μg kg^−1^) for *zanzibariensis*. Differences in toxicity are assumed to be based on variations in the isotoxins of ricin expressed by the different seed types. Subsequent analysis of ricins extracted from different castor seed cultivars, using 2D polyacrylamide gel electrophoresis and silver staining, has revealed differences in the range of ricin toxins expressed, based upon isoelectric points [10]. At supralethal doses, an inverse relation has been found between times to death and Ct [9], varying from about 36 h at higher Cts (for Hale Queen ricin, 119.3 and 11.9 mg min m^−3^) to 66 and 138 h at lower Cts (Hale Queen, 4.54 and 5.96 mg min m^−3^). A similar Ct: time to death relationship was found for *R. zanzibariensis* and also for abrin, in which the LCt_50_ was found to be 4.54 mg min m^−3^ (estimated LD_50_ 3.3 μg kg^−1^).

Particles of around 1 μm would be expected to flow with the airstream around obstacles such as the nasal turbinates, rather than impact through inertia, and reach the deep lung. Roy *et al.* [11] examined the comparative distribution of inhaled aerosolized ricin particles of different size ranges of 1 μm and 5-12 μm MMAD. This study showed that in mice exposed nose-only to aerosol, ~60% of the 1 μm ricin particles were deposited in the lungs, followed by the trachea, with very small amounts (~1-2%) being measured (ELISA) in nares and stomach. At the larger particle sizes, most ricin was found to be in the trachea, with less in the lungs (~20%); very little ricin was found in the nares (1-8%) and stomach. It was rather surprising that so little of the larger sized ricin particles were filtered out by the nares. The authors felt that the heterogeneous size distribution of the larger particles and poor control of the dispersion of particles, partly due to restrictions imposed by working in Class III safety cabinets, may have influenced regional distribution patterns and subsequent ricin distribution rates. The influence of particle size on lethality was marked, in that those animals which received a supra-lethal dose of 1 µm particles of ricin died at 72 h post-exposure, whereas those receiving a similar challenge of larger particles (>5 µm) all survived.

The body distribution of inhaled ricin was examined by Deobler *et al.* [12]. They exposed mice nose-only to aerosolized (1 μm MMAD) ^125^I-ricin and found radiolabel in the lungs but also in the gastrointestinal tract, although release into the circulation was found to be minimal. It is reasonable to assume that some inhaled aerosol would be swallowed, although it is unlikely that it would contribute significantly to the lethality of the total dose received since the oral toxicity of ricin is very much lower than that of the inhaled toxin. In the mouse, the inhaled LD_50_ was cited as being between 3 and 5 μg kg^−1^ whereas the comparative lethality by ingestion is 20 mg kg^−1^ (6). 

The largest quantity of ^125^I-ricin was found in the lungs, being highest at 15 min post-exposure, declining by around 50% in the first four hours after exposure and then dissipating from the lungs much more slowly between four and thirty hours, thereafter. Radioactivity was also measured in the stomach, spleen and liver; it was also found in the blood, with as much as approximately 600 pg of ricin equivalent label being measured here at times between one and eight hours pose-exposure.

Inhalation toxicity studies of ricin have been undertaken by other laboratories where the mouse was the small animal model of choice, although different strains have been used. For example, Balb/c mice were challenged by inhalation using whole body exposure to pure ricin (variety of origin not given) [13] and an LD_50_ of 11.2 μg kg^−1^ was determined, which agrees well with the data we found in inhalation studies in the rat for ricin extracted from *R. zanzibariensis*. Differential susceptibilities to inhaled ricin were also mentioned in relation to mouse strain and the LD_50_ for inhaled ricin in the most sensitive strain, BXSB, was approximately 2.8 μg kg^−1^. It was also intimated by these authors, that the toxicities of different sources of ricin can vary, an observation which we made from our own inhalation toxicity studies in the rat between ricins originating from Hale Queen and *R. zanzibariensis* (above). 

Recently, ricin isoforms of similar molecular weights, were purified using a standard approach [14] and resolved using lactamyl-sepharose to reveal molecules of very different toxicities using *in vitro* (in Vero cells) and *in vivo* (in mice) techniques [15]. One isoform, RIII, representing 35% of the ricin isoforms in the seed [16], was substantially more toxic (4-8 fold) than the others; the presence of a higher proportion of this isoform in ricin produced by an cultivar of *Ricinus communis*, could go some way to explain the different toxicity values measured for Hale Queen and zanzibariensis. It was also the most heavily *N*-glycosylated isoform, bearing 22 glycopeptide fragments compared with whole ricin, which was found to contain 25; the sugars were mainly hybrid/complex type with mannose, as the hexose sugar units [16]. 

A very recent study by Benson *et al.* [17] examined the acute toxicity of ricin, associated histopathology, deposition and clearance kinetics in rats and mice. The study determined that the estimated mean lethal dose for ricin in rats was 0.24 μg kg^−1^ and for mice, 0.58 μg kg^−1^ with survival times ranging from ≤2 to 6.2 days in rats and from 2 to 7 days in mice, depending on dose (shorter times to death as concentration increased). These lethality figures suggest a potency of ricin some 15-40 times higher in the rat depending on the source of ricin (from Hale Queen or zanzibariensis) and are between 5 and 19 times higher in the mouse than figures determined before [9,14]. This may be in part due to the calculation of deposited dose in this study [17] which involved the incorporation of a “deposition fraction” into a calculation combining aerosol concentration (C), minute volume of the animals (MV), exposure time (t), (µg ricin deposited = µg ricin/L air (C) × MV × t × deposition fraction. The latter was derived by Schlesinger in 1985 [18]. Our observations [9] were based on the concentration (C) of ricin aerosolized, time of exposure (t) and inhaled using minute volumes (MV) without the introduction of a deposition fraction. 

In a recent ricin inhalation study in mice [19], we used a head only delivery of 1 μm MMAD aerosol particles and measured ricin by ELISA in tissue extracts. We found majority of the toxin to be present in the lungs, smaller quantities of ricin in the liver but also small amounts in the heart, kidney and some lymphoid tissues. No ricin was at any time found in the blood or gastro-intestinal tract using the ELISA. It is possible that Doebler *et al.* [12] were picking up radio-iodine rather than ricin in the blood and that, in our study, swallowed and denatured ricin was not picked up by the antibodies used in our ELISA. 

### Histopathology of Intoxication

Histopathology resulting from the aerosol exposures of rats to ricin extracted from Hale Queen and *R. zanzibariensis* cultivars of *Ricinus* communis plants confirmed that ricin particles reached the deep lung. Animals were exposed (and culled over a time course of up to 14 days) by inhalation to aerosols of either type of ricin of particle size <1 μm at similar Ct levels (LCt_30_). The pathogenesis of damage resulting from the inhalation of aerosols of both varieties of pure ricin were broadly similar and were found to be confined to the lungs [9]. At two days post-exposure, pathological changes to the lung included necrosis, as well as apoptotic deletion of the epithelia of the bronchi, segmental bronchioles and the terminal bronchioles. A well established acute alveolitis was present with severe interstitial (perivascular) and intra-alveolar oedema, with an excess of apoptotic body formation. Macrophages were present within the interstitial alveolar septae. The upper airways were lined by intact, non-inflamed epithelium and extra-pulmonary organs were histologically normal. 

At three days post-exposure, the dominant feature was an overwhelming diffuse alveolar oedema, established acute alveolitis with severe capillary congestion and infiltration of the pulmonary interstitium by large activated macrophages. Careful examination also revealed small focal, sometimes florid areas of type II pneumocyte hyperplasia. The large airways continued to demonstrate necrosis of the lining epithelia but there was also evidence of early epithelial regeneration. By four days the histological picture was dominated by rapidly resolving pulmonary oedema and general pulmonary consolidation by small lymphocytes, activated macrophages and a striking proliferation of type II pneumocytes. By this time, the bronchioles and bronchi were completely re-epithelialised with lumina free-from oedema fluid, which had been present at two and three days. In keeping with the significant consolidation of the lung parenchyma (and hence an increase in pulmonary vascular resistance), the solid peripheral organs all showed severe passive venous congestion. By seven days post-exposure, multifocal but extensive islands of persistent consolidation were apparent, with all features described above at four days. Between the islands of consolidation, the lung tissue approached a normal appearance with complete absence of interstitial/intra-alveolar oedema and residual acute/chronic alveolitis.

Fourteen days after exposure to ricin, all animals appeared to be essentially normal, except for a few focal areas of persistent intra-alveolar foamy (lipid-laden) macrophage infiltration.

In essence, ricin damages the pulmonary epithelial cells which form part of the alveolae, causes inflammation, which leads to pulmonary flooding with oedema fluid; if damage is severe (if the dose of ricin inhaled is large enough), the victim effectively drowns. Damage was limited to the lung and the other organs examined remained normal in appearance. 

Using electron microscopy, Brown *et al.* [20] observed the initiation of oedema formation, initially perivascular, from 12 h following inhaled ricin aerosol (LCt_30_). The situation progressed from 15-36 h to include interstitial and intra-alveolar regions. Additionally, inflammatory cell infiltration was evident from 12 h post challenge but the greatest numbers of macrophages and polymorphonuclear leucocytes were noted between 24 and 48 h.

The epithelia of both the lower respiratory tract and the alveoli were subject to destructive inflammatory damage, with evidence of necrotic and apoptotic processes. Signs of repair were also noted in the form of hyperplasia of type II epithelial cells. Beyond 96 h, a gradual clearing of oedema fluid was observed although complete resolution of damage was not achieved until 14 days post exposure. 

The study by Benson *et al.* [17] also observed deposition in the nasal cavities and respiratory tracts of the rodents, where the increasing dose of ricin in rats was accompanied by more damage. In mice, greater damage in the tract occurred at later times as the lesion had more time to develop. In this study, the degree of pulmonary oedema was not apparently as great as that reported before [9], although inflammatory pulmonary lesions were observed as in the earlier studies. However, these lesions were more severe in animals which survived longer [17], and type II alveolar hyperplasia was noted in the repairing lungs. This study also reported thymic lesions (thymocyte apoptosis in rats and atrophy secondary to loss of cortical thymocytes in mice dying from ricin exposure) and splenic neutrophilic inflammation accompanied by lymphocytic and haematopoietic cell apoptosis. 

Wong *et al.* [21] instilled doses of ricin, which were very high (supralethal) or sub-lethal (2-5 μg·100 g^−1^), into the tracheae of mice and observed effects on pulmonary and extra-pulmonary tissues. At the higher dose (20 μg·100 g^−1^), histopathological lesions were observed after 48 h and included perivascular and peribronchial oedema, disruption or denudation of the bronchial epithelium with evidence of haemorrhage, neutrophilia in bronchial and alveolar areas and apoptotic bodies. Fibrin was found to have deposited abundantly throughout the lungs of mice following ricin exposure (20 μg·100 g^−1^), in particular within the microvasculature of the alveolar septa. Typically, lesions were seen to be patchy, consistent with instillation, as compared with the more uniform distribution seen after inhalation of ricin. Proinflammatory transcripts ERK and JNK were activated (phosphorylated) in bronchial and bronchiolar epithelial cells, alveolar epithelial cells and endothelial cells (using immunoblotting for phospho (p)-ERK, p-JNK, p-p38 MAPK and p38 MAPK in lung wash lysates or immunohistochemical staining in lung sections of control or ricin-treated mice); a commensurate activation of NF-κβ was observed in most lung cells 48 h after ricin instillation. The presence of the apoptotic marker, caspase 3, was found throughout the lungs in patches of airway cells and scattered cells in the alveolar parenchyma in mice exposed to ricin. Microarray analysis confirmed a ricin-induced elevation of cytokines and chemokines (TNF-α, IL-6, IL-1β, CXCL1 and CCL2) in mouse sera, as well as increased expression of proinflammatory transcripts in lung and other tissues. Of particular interest was the finding that 28S rRNA lesions could be detected in kidney tissue and other organs (lungs, spleen, liver and blood) taken from ricin-instilled mice and a mild accumulation of inflammatory cells after instillation of 20 μg·100 g^−1^ ricin. Impaired kidney function (implied by the appearance of serum albumin in urine and increased blood urea nitrogen) accompanied the glomerular pathology observed. Observations indicated that instilled ricin accessed the circulation and it was shown that the same dose administered into the oesophagus (ingestion) did not access the circulation or other organs. This study is of interest in that it suggests that intra-pulmonary ricin does enter the blood and causes damage in other systemic organs. It is important to note that the doses of ricin administered were very high indeed; the so called “sub-lethal” dose of 2-5 μg·100 g^−1^ translates to 20-50 μg kg^−1^. We instilled 2 μg kg^−1^ of ricin into rats and found it to be supra-lethal [22]. The higher dose used in this study (20 μg·100 g^−1^) would translate to 200 μg kg^−1^, which is an extremely high dose of ricin; this might explain why damage to remote tissues was observed following administration of such a quantity of the toxin. 

The United States Army Medical Research Institute of Infectious Diseases is one of very few institutes to have undertaken inhalation toxicology studies with ricin in non-human primates. They studied two non human primate species, the rhesus monkey and the African Green monkey; lethal doses of ricin were determined to be 15 and 5.8 μg kg^−1^, respectively, using the staircase method [13]. These inhalation LD_50_ estimates are of similar order to those found in the rat for ricin derived from *R. zanzibariensis.* Recent information has been obtained [23] that indicates that the LD_50_ by inhalation of ricin (pure ricin extracted from *R. zanzibariensis*) was around 5 μg kg^−1^ in the rhesus macaque, which was close to the figure obtained for the African Green monkey. Histopathology was examined at death (36-40 h) following inhalation of supralethal doses of ricin aerosol (approximately 21 to 42 μg kg^−1^) in the rhesus monkey [24] and found to resemble closely that seen in the rat. Lesions were confined to the thoracic cavity with diffuse necrosis and acute airways inflammation with alveolar flooding and peribronchovascular oedema. Inflammation was also widespread in the trachea, the pleura and mediastinal lymph nodes. Similar findings in the African Green monkey were reported by Wannamacher *et al.* [13] where the lungs from ricin exposed animals exhibited haemorrhagic congestion and moderate multifocal bilateral oedema. Pathology was confined to the lungs and included fibrinohaemorrhagic pneumonia, alveolar and sometimes bronchiolar oedema, with necrotic changes to alveolar and bronchiolar epithelia. The authors concluded that the consistency in pathology between rodents, other reported species and non human primates adds confidence in predicting a similar picture in human victims of inhaled ricin. 

The major problem caused by the inhalation of these toxins seems to be pulmonary flooding of the lungs resulting from primary damage to epithelial and, presumably, underlying blood vessel endothelia. Damage is probably exacerbated by the massive inflammatory response mounted by the host to the presence of the toxin and the primary damage caused. Together, these events lead to respiratory insufficiency which, in severe cases, leads to death.

There are no human cases of inhaled ricin to report but studies in rodents and non-human primates would indicate that symptoms of poisoning by this route of intoxication would be a gradual feeling of lassitude and loss of interest in the surroundings. Respiratory symptoms including wheezing and increased difficulty in breathing and tightness of the chest as lungs became oedematous would gradually increase from 24 h (after a fatal dose of ricin), with loss of appetite. Death occurs with anoxic convulsions, when it is impossible to obtain sufficient oxygen for brain function. Overall, ricin is very toxic by inhalation of particles small enough to reach the deep lung, where it causes disruption to alveolar epithelial membranes leading to pulmonary flooding and inflammation; this is a constant feature across species. Whether inhaled ricin enters the blood in sufficient quantity to cause damage in peripheral organs is still uncertain. Understanding the consequences of exposure to ricin by this route is very important in attempting to limit damage and prevent lethality.

## 3. Toxicity of Ricin by Parenteral (Intramuscular) Administration

Although ricin poisoning by intramuscular, subcutaneous or intravascular routes are relevant to military and terrorist attack scenarios these have been poorly investigated. Ricin could certainly be administered on a larger scale by skin penetration and into muscle, by explosive devices containing sharp materials such as flechettes coated with the toxin. Then, the introduction of the toxin into the body would probably involve a mixture of the three routes listed. On an individual basis, Georgi Markov was murdered in 1978, by the introduction of a toxin, probably ricin, from a small hollow pellet fired into his leg from a gas gun which was disguised as an umbrella [25,26]. Symptoms included pain at the injection site of the pellet followed by nausea, vomiting, fatigue and fever developing over the 24 h after injection. Gastrointestinal haemorrhage, hypovolaemic shock and renal failure developed subsequently and Markov died three days after being shot with the pellet. 

In studies on intramuscularly-administered ricin, we found that the LD_50_ by this route was between 3 and 5 μg kg^−1^ in the rat, with death occurring at between 36 and 48 h. We have also investigated the fate of radio-iodinated ricin administered intramuscularly into the thigh muscle of rats [27]. In interpreting distribution of ricin by virtue of this radiolabel, the same reservation of a possible separation between some label and ricin must be borne in mind. During intervals over a 30 hour period following administration, radioactivity was found to accumulate in lymph nodes draining the muscle, in the liver and other tissues. Using ELISA, it was possible to identify ricin in the lymph nodes and liver [28] and the toxin could be localised in these nodes using immunocytochemistry [29]. Closer examination of the lymph draining process suggested that ricin reached the lymph node an hour or so after injection and was bound to different cell types including lymphocytes by a mechanism which could be inhibited, *in vitro*, by lactose [30]. Damage to the small intestine was a feature following i.m. poisoning with ricin, with the infiltration of plasma cells and activated macrophages into the lamina propria and apoptotic deletion of these and structural cells in the ileum [31]. 

When ricin is administered into the body in this way, and is able to enter the blood circulation, then it potentially has access to all systemic organs. The distribution of ricin would have a similar fate to that administered intravascularly but the time to effect would be expected to be slower following intramuscular administration. 

Ramsden *et al.* [32] observed the toxicity, distribution and excretion of ricin in rats, focusing on ^125^I-labelled ricin. After intravascular administration, radioactivity distributed rapidly by 0.5 h to the liver (46%) and spleen (13%), although the concentration per g of tissue was highest in the spleen (33% of injected dose/g, 4 times that found in the liver). Loss of ricin from the liver was rapid (from about 40% of injected dose to less than 10% in 6 h). Radiolabel was detected also in the bone marrow, lung, kidney, heart, gut, lymphoid tissue, blood and muscle. Excretion of material was primarily via the kidneys in the urine and approximately 70% of the initial radioactivity was detected here within 24 h, present in freely dialysable metabolites of ricin. Faecal excretion of ricin only accounted for about 2% of the injected dose. 

### Histopathology of Intoxication

Histopathology of ricin poisoning by the intramuscular route has not been very extensively investigated. Information is bolstered by evidence from the only reported case of human poisoning, that of Georgi Markov [25]. From the pellet which was introduced into his thigh muscle, ricin was distributed around his body, and many tissues were affected. Damage included the presence of severe local lymphoid necrosis (in the inguinal lymph nodes which drained the injection site), liver necrosis, diffuse inflammation of the kidneys and spleen. Haemorrhagic necrosis of the small intestine was also noted as well as the pancreas and testes. The myocardium and conducting tissues were found to have petechial haemorrhages which also featured in adrenals, gut wall and lymph nodes. A mild pulmonary oedema observed was thought to have been secondary to cardiac failure. 

The key feature of toxicity via the intramuscular/intravascular route is the fairly rapid distribution of toxin around the body to most organs, making therapeutic treatment a challenge. 

## 4. Ingestion of Ricin

Oral toxicity of ricin has also been relatively poorly studied. In humans, there have been many attempts at suicide or inadvertent poisoning through eating castor oil seeds. 

Much more ricin is required to achieve lethality by the oral route as the toxicity of ricin when delivered this way is much lower than by inhalation and parenteral delivery routes. It has been noted that in the relatively few studies of oral intoxication by ricin, the precise means of dosing with the toxin (e.g., by mouth or by gavage) have been omitted. In the mouse the LD_50_ for orally administered ricin is quoted by Franz and Jaax [6] as 20 mg kg^−1^ bodyweight with a time to death of approximately 85 h. The lower toxicity by this route is probably a reflection of the poor absorption of ricin from the intestine. Kumar *et al.* quoted an LD_50_ value for male Swiss Albino mice [33] of 28.29 mg kg^−1^. In another review by Audi *et al.* [34], the LD_50_ of ricin by the oral route was reported to be 30 mg kg^−1^ and a human LD_50_ estimate was given as 1-20 mg ricin kg^−1^ bodyweight. 

While there have been many reported suicide attempts through the ingestion of castor oil seeds, there is great difficulty in the interpretation of the toxic dose because of variables including differences in ricin content of seed types (e.g., geographic region of plant growth, time of harvesting seeds, degree of hydration of seeds and ricin isotoxin content) size and weight of seeds ingested as well as the degree of mastication to release the seed contents, including the water-soluble ricin. 

Victims of oral ricin poisoning feature prominent gastrointestinal symptoms which may include colicky abdominal pain, vomiting, diarrhoea, heartburn and oropharyngeal pain. In addition, haematemesis and melena (black tarry faeces associated with gastrointestinal haemorrhage) are less commonly-reported symptoms. Fluid loss may result in electrolyte imbalance, dehydration, hypotension and circulatory collapse [33]. Associated symptoms may include tachycardia, tachypnoea, sweating and peripheral cyanosis [35]. 

A small study on the ingestion of ricin in Balb/c mice was undertaken in our laboratory which served as a control for a larger experiment. Groups of three mice were administered (by gavage) 10 or 20 mg kg^−1^ of ricin purified from *Ricinus zanzibariensis* seeds. Animals appeared normal over a 7 day period following oral dosing with 10 mg kg^−1^. Forty-eight hours after dosing with 20 mg kg^−1^ ricin, however, all mice were relatively inactive, they had lost the inquisitive habit typical of rodents and their fur was piloerected. One animal died on the fourth day and the stomach and small intestine showed evidence of inflammatory reddening of the small intestinal wall, close to its point of exit from the stomach. From this very limited study, the LD_50_ of pure ricin (from *Ricinus zanzibariensis*) in the mouse concurred well with other reported data [6], at around 20 mg kg^−1^ (unreported observations). 

Ishiguro *et al.* [36] administered ricin (10 mg kg^−1^) orally to rats, mixed into olive oil (precise method not given). About 40% of the administered ricin moved to the large intestine from the stomach within 12 h where it remained for at least 72 h, about 20% being excreted in the faeces. Ricin was found in lymph after 1 h, but entered the plasma, largely intact, more slowly, being maximal at around 4 h. Transfer to the blood was probably a consequence of the damage inflicted on the intestine by the toxin. Using enzyme-immunoassay, ricin was detected in liver at 6 h, increasing over 48 h when it peaked at 95 ± 20 μg/organ. Ricin appeared in spleen after 3 h and was maximal at 6 h (0.1 ± 0.025 μg/organ), remaining constant to 72 h. Ricin could not be measured in pancreas, kidney, lung, heart and brain. 

Godal *et al.* [37] investigated the opportunity of radioimmunoassay to detect ricin (and abrin) in the blood and achieved a limit of detection of 50-100 pg mL^−1^ with the inconvenience of having to deal with radioactive labels. Cook *et al.* [38] used a sensitive ELISA for ricin (or ricin-equivalent material, *i.e.*, peptides or degraded ricin) in samples taken at 24 h after poisoning with 8 mg ricin/kg in the Porton Wistar rat. Ricin was detected in blood, in association with the cellular fraction (1.4 ng per 2 mL sample), in liver tissue (9.5 ng g^−1^), gut (stomach plus proximal duodenum; 31.7 ng g^−1^), spleen (39.6 ng g^−1^) and kidney (10.9 ng g^−1^). In the case of the kidney, the signal was not significantly different from the corresponding vehicle assay. Comparison between the findings of this study with those of Ishiguro *et al.* [36] are qualitatively similar but quantitatively, levels of ricin (equivalent) detected would seem to be much smaller, reported in ng/g rather than μg/organ.

Essentially, ingested ricin finds its way fairly rapidly into the blood and then to other tissues within a few hours of poisoning. 

### Histopathology of Intoxication

Histopathology of lethal cases of oral ricin intoxication includes multifocal, often severe, ulceration and haemorrhage of the gastric and small intestinal mucosa accompanied by necrosis of the draining mesenteric lymph nodes, gut associated lymphoid tissue and spleen. Necrosis is observed in liver tissue, especially the reticulendothelial Kupffer cells and diffuse inflammation of renal tubules (nephritis) and the spleen [6]. Apoptotic cell death is also cited in the histopathology of ricin toxicity by ingestion [34].

## 5. Detection of Ricin

The detection of ricin in samples, including body tissues and many other materials, which may necessarily be examined has followed several lines of investigation. Clearly, it is most important to determine whether a person has been exposed to a chemical/biological agent in order to initiate appropriate therapeutic medical treatment, but it is also necessary to forensically determine exposure in order to attribute blame, from a legal viewpoint. It is also very much in the minds of various authorities that ricin might be used by terrorists to contaminate foods; it is, therefore, necessary to have the technical means to demonstrate whether such an action has been undertaken. From a defensive stance, there has been an upsurge in the development of assays to identify ricin and other toxins of concern, in body tissues and foods, but also in other matrices. Many approaches have been based on antibody recognition, with a number of strategies applied to increase sensitivity; these have been packaged in a variety of formats including the enzyme-linked immunosorbent assays (ELISAs), with various means of visualising the target, lateral flow devices, immunoaffinity columns or surface plasmon resonance (resonant mirror devices). A second approach, particularly applicable to instances where crude ricin preparations are involved, is the use of PCR to detect DNA or RNA relevant to the toxin or its plant origins. A third approach has been that of mass spectroscopy, for the identification of ricin or peptide components of the holotoxin. A fourth approach is that based on ricin activity, but such techniques are not specific to ricin and are shared with other ribotoxins. These approaches will be explored in more detail below.

### 5.1. Enzyme-Linked Immuno-Absorbent Assay (ELISA)

ELISAs to detect ricin have been around for at least 30 years and were first developed to replace the rather more cumbersome method of radioimmunoassay with the obvious hazard of dealing with radioisotopes [37]. The ELISA-based methods replaced radioimmunoassay and were developed in the 1980s, with quite good limits of detection; for example, with a limit of detection for ricin of 2 ng·100 μL^−1^ of sample [28] which was superseded in our group by a method which had been made more sensitive through increasing the amount of enzyme linked to the target complex by the use of avidin-biotin immunoperoxidase complex in a “sandwich” of reagents [39]. This step increased the limit of sensitivity of the assay 100 fold, enabling us to detect 20 pg·100 μL^−1^ in tissue extracts from rats poisoned with ricin, which had been treated to release the ricin for assay. This “sandwich” ELISA was used more recently to examine the fate of ricin following intratracheal instillation and ingestion [38]. Poli *et al.* used a similar approach [40] in human urine and plasma to quantify levels of ricin spiked into these matrices. They achieved limits of detection of around 100 pg ricin·100 μL^−1^ which could be improved to 10 pg·100 μL^−1^ by increasing amounts of biotinylated antibody and avidin-linked enzyme in the assay, with good coefficients of variation; they also formulated a chemiluminescent assay with a similar performance. The “sandwich” ELISA format was employed [41] to detect ricin, also spiked into human urine and serum; here ricin was captured by an antibody raised against ricin B chain and reported by a monoclonal antibody directed against ricin A chain conjugated with peroxidase. This assay was reported to have a limit of detection below 5 ng mL^−1^ in buffer, and in a 10 fold dilution of urine or 50 fold dilution of serum. This format is still favoured for the analysis of ricin in animal tissues following poisoning [42]. 

The detection of ricin in food, specifically ground beef, was achieved using electrochemiluminescence (ECL), which basically follows ELISA methodology, but the reporter antibodies were tagged using streptavidin with Ru(II) tris-bipyridine 4-methylsulfonate for an indirect assay [43]. ECL exploits multiple excitation cycles to amplify the luminescent signal and improve sensitivity. The mechanism of excitation and the relatively long emission wavelength (620 nm) potentially provide resistance to matrix effects, beneficial when interrogating complex materials such as fatty foods. Compared to ELISA, although the limits of detection were similar for both assays (0.5 ng ricin g^−1^ for ECL and 1.5 ng mg^−1^ for ELISA), the ECL offers a wider dynamic range of operation, a lower coefficient of variation and less interference with complex matrices such as foods; the signal to noise ratio for the ECL was as much as five-fold better than the ELISA. 

The sensitivity of the ELISA-based assay format has been much improved by technical modifications, to enhance the limits of detection through amplification. The use of biotinylated reporter antibodies with avidinylated enzymes has already been shown to improve assay sensitivity 100 fold (39), but the combination of polymerase chain reaction (PCR) to amplify a DNA-labelled reporter system in place of an enzyme, is reported to achieve enhanced limits of detection into the femtogram per millilitre in human serum [44]. However, when this method was applied to the challenge of detecting ricin in complex food matrices [45], although a detection limit of 10 fg mL^−1^ was achieved for ricin in PBS buffer, the limits were 10 pg mL^−1^ in liquid egg and milk and 100 pg mL^−1^ in ground beef. These are clearly superior to the ECL and ELISA methods. The same group used the immuno-PCR assay to detect ricin in murine sera and faeces and reported a limit of detection of 1 pg mL^−1^ based on linear regression and 95% confidence interval [46].

### 5.2. Immunochromatographic Test Strips (Hand-Held Assays)

Hand held assays are based on ELISA technology but use a lateral matrix, usually nitrocellulose, carrying an immobilised detection antibody across which flows a colloidal gold-labelled reporter antibody. Shyu *et al.* [47] reported the development of a rapid immunochromatographic test (ICT) for the detection of ricin, comprising two monoclonal antibodies, one directed against ricin B-chain and immobilised on the nitrocellulose and the other directed against ricin A-chain, labelled with nanometre sized colloidal gold particles. Sample solution for interrogation is added to an absorbent area, where it mixes with the gold-labelled anti A-chain antibodies. If ricin is present, it combines with this monoclonal, forming a complex, and this is captured by the immobilised anti B-chain antibody, where a visible red line develops. This assay could detect 50 ng mL^−1^ of ricin in 10 min and the limit of detection can be increased up to 200 times following silver enhancement. An ICT was reported [48] for the diagnosis of inhalational ricin poisoning; this test has a rapid response time and a limit of detection of 1 ng mL^−1^. It was used to screen ricin samples prepared from 19 different cultivars or *Ricinus communis* and found to report them all equally well [49]. We have been examining the potential use of ICT for the diagnosis of ricin poisoning using body fluid samples from mice which have been exposed to ricin by inhalation. The tests do show some promise and, moreover, in comparison with the analysis of the samples using ELISA, ICT can be used semi-quantitatively if scanned using a densitometer and read against standards, often with good agreement with ELISA data. This study is soon to be reported.

### 5.3. Immunoaffinity Columns

The immunoaffinity column (IAC), which is also based upon antibodies for detection and reporting, functions like an ELISA in a column matrix. This approach offers the advantage of being able to cope with more dilute solutions of target antigen, as they may be applied to the column and absorb the target which builds up to a detectable concentration. The technique has been applied to the detection of different microorganisms and several toxins, but no reports have yet appeared for ricin detection. IAC have, however, been reported to have a sensitivity of one mouse lethal dose per mL for botulinum C and D in culture supernatants [50] and was found to detect 1 mouse lethal dose 50 per mL of botulinum A in a 5 mL sample of human serum [51]. In this latter report, the IAC test was also compared with an ICT to detect the same antigen, which was sufficiently sensitive to detect a human lethal dose of botulinum, but the IAC offered the advantage of being able to concentrate antigen if present in low concentration. Others have reported the application of the IAC to botulinum neurotoxins and *E. coli* 0157 in foods [52] and to the detection of *Francisella tularensis* in human samples (blood and urine) [53]. We are also applying this approach to the sensitive and rapid detection of ricin in body fluid samples with a view to making an early diagnosis of poisoning. 

From what we have seen in our observations, ICT offers potential for rapid diagnosis of ricin in a short time (<30 min) and is sensitive down to ng quantities. This rapid detection is very important because it is necessary to commence therapeutic strategies quickly, certainly within a few hours of exposure. The IAC technology is also very sensitive, again down to low nanogrammes, is fast (an answer in <1 h) and offers the advantage of being able to capture target in low concentration, by passage of several millilitres of sample through the column. Both techniques, therefore, have their merits in the defensive battery of tests leading to rapid diagnosis and speedy commencement of therapy for those exposed to the toxin. 

### 5.4. Surface Plasmon Resonance

The final antibody-based technique to be mentioned here is surface plasmon resonance (SPR). In this technique, a thin layer of gold on a glass or quartz surface forms a mirror to reflect a laser generated light beam. Antibodies specific for ricin, for example, are adsorbed onto the gold surface. The angle of reflection of the light beam changes subtly when ricin in a solution travels past the immobilized antibodies, when they capture their target. The reflection is influenced by oscillation of electrons (plasmons) in the gold layer which are excited by the beam of light, causing less light to be reflected. When immobilised antibodies bind ricin, the refractive index of the gold surface changes; this alters the resonant frequency of the plasmons, which in turn further alter the amount of reflected light. In essence, the detection is based on the fact that the adsorbing molecules cause changes in the local index of refraction, changing the resonance conditions of the surface plasmon waves. The method is very sensitive and the change in refractive index measured is directly proportional to the mass of the target molecule bound [54,55,56].

A whole range of toxins including Aflatoxin B1, Ochratoxin A, Fumonisin B1, Saxitoxin, Staphylococcal enterotoxin B, Tetanus toxin, and ricin or ricin A chain have been detected using SPR [57]. Rapid detection of ricin, at a concentration as low as 0.5 ng/mL [58], was achieved with a panel of immobilised monoclonal antibodies to detect ricin extracted from six horticultural variants. Portable SPR instruments are being developed for use in the field. One such instrument [59] could detect ricin at 200 ng/mL in 10 min. From a defensive angle, the use of SPR was engaged to screen food for the presence of a number of toxins including ricin as well as those which are common bacterial food contaminants [60]. The technique can also be used as a research tool and, in the case of ricin, has examined the binding properties of ricin and its B-chain (association constants) with immobilised glycolipids of varying chain length in phospholipid membranes [61]. SPR has also been used to investigate the interactions of carbohydrate inhibitors with Shiga-like toxin, a ribotoxin with a similar mechanism of action to ricin [62]. SPR is one of the assay technologies applied to the monitoring of the atmosphere for biological agents on a concentrated solution from continuous air sampling. Much emphasis is, therefore, placed on antibodies in the detection of threat agents. Generally speaking, antibodies form an invaluable tool both for the identification of ricin but also, they may be used therapeutically to mitigate the effects of ricin following exposure. One limitation of antibody-based techniques is that if a monoclonal antibody is employed, only one epitope is specified and this may be absent in the sample; polyclonal antibodies get around this problem but the chance of cross-reactivity with other antigens is increased, possibly making detection less specific. Further, antibody-based techniques do not necessarily provide an indication that the toxin is active and Kumar *et al.* [63] have given examples where denatured ricin can still be detected by antibody-mediated tests. 

### 5.5. Polymerase Chain Reaction

The isolation of ricin DNA from liquid egg and milk spiked with a ricin/acetone powder preparation, was achieved most effectively using acetyl trimethylammonium bromide and amplified using the real time polymerase chain reaction (RT-PCR) [64]. This shows that molecular biological methods can be applied to tests for ricin contaminated foods as counter-terrorist approaches. 

In a real situation with a “white powder” found in a hotel room in Las Vegas, RT-PCR was one of the methods which were successfully applied to identify ricin [65] in 2008; the test was supported using mass spectrometry. Others have reported the use of field-based RT-PCR [66] for the detection of ten threat agents, including ricin, without the need for sample extraction or purification.

Other related studies using molecular biology have included an analysis of nucleotide polymorphisms associated with variants of the *Ricinus communis* plant grown around the world [67]. Genetic diversity amongst this group of plants was also examined by considering the genomes of seven diverse cultivars and comparing the data to a reference standard of a widespread cultivar, Hale [68]. The study focused on single nucleotide polymorphisms (SNPs). 

RT-PCR was also used in conjunction with immunodetection (see above) to enhance the limits of detection of ELISA type approaches using a DNA label in place of an enzyme. 

It is important to remember that RT-PCR will only be a viable option where crude ricin preparations are concerned. If ricin is highly purified, then it is very unlikely that there will be any nucleic acid material remaining in the preparation. However, since the internet recipes would all produce crude ricin, then this method of detection may offer additional benefits. 

### 5.6. Mass Spectroscopy

The advantage of mass spectrometry in the identification of ricin is that it is based on physico-chemical properties of the protein itself and does not rely on antibodies. Providing that a fairly clean preparation of ricin can be made, this approach provides strong orthogonal evidence to support the identification of the toxin. 

Ricin has been characterised by electrospray mass spectrometry, capillary electrophoresis and surface plasmon resonance by Despeyroux *et al.* [69], using whole ricin molecules, as a means of studying the heterogeneity of ricin extracted from several different cultivars of *Ricinus communis*. However, the evidence provided is not conclusive identification of the toxin because it did not provide information linking with structural peptide backbone data. Fredriksson *et al.* [70] added this important development in their study by providing information on mass spectrosocopy of ricin at the amino acid and glycopeptide level. Ricin was prepared from seeds of several cultivars, denatured, reduced, digested by trypsin and analysed by high performance liquid chromatography followed by electrospray mass spectrometry using a Q-TOF tandem mass spectrometer. Ricin samples produced three ricin containing fractions after column chromatography (cation exchange) except for *R. zanzibariensis*, which produced only one ricin peak. After digestion and mass spectrometric analysis, sequences were compared to an amino acid sequence database. This analysis enabled ricin-specific trypsin digest peptides to be selected from the A and B chains of ricin which would enable the identification of ricin to be made in a single analysis run. 

Ricin enzymic activity was reported in a single step method with minimal sample preparation [71] which measured adenine release from DNA using real time mass spectrometry. Becher *et al.* [72] combined immunoaffinity capture using a ricin B chain-specific monoclonal antibody coupled to magnetic beads followed by the determination of adenine released by the active toxin from an RNA template by liquid chromatography tandem mass spectrometry (LC-MS/MS). The method, which was tested in milk, tap or bottled water, incorporates specificity and provides information on toxin activity with a limit of detection of 0.1 ng/mL with a low variability (10%). Although the full assay with this low limit of detection took twenty six hours to complete, a less sensitive response (3-fold lower) could be achieved in six hours. 

Kalb and Barr [73] described a three phase method to identify ricin in food or clinical samples (milk, apple juice, human serum or saliva). The approach comprised of the capture of ricin using immunoaffinity technology with antibody coated beads, a measure of activity of the toxin by probing depurination of a DNA substrate (using matrix-assisted laser desorption /ionization time-of-flight mass spectrometry; MALDI-TOF) and the analysis of tryptic peptides using LC-MS/MS. Together, these three components provided a sensitive and selective analysis and activity determination of ricin recovered from food or clinical samples. Another laboratory described a multiplex approach to the detection of microbial and plant toxins [74] in complex sample matrices, in particular, with foods in mind (milk, apple juice and orange juice, ham and bacon rind). The method employed the affinity capture approach using toxin-specific antibodies coupled to paramagnetic beads, followed by trypsin digestion and the identification of toxin-specific fragments using MALDI MS. The approach identified ricin, SEB, Botulinum neurotoxins A and B, but further differentiated A1, A2, B, B1 and non-proteolytic B subtypes. 

At Dstl, ricin was detected in urine collected at between 8 and 12 h following exposure, in a murine model of intoxication by the intravenous route. Urine samples were separated using one-dimensional gel electrophoresis. Three key peptide sequences matching ricin and/or *Ricinus agglutinin* were subsequently identified by mass spectrometric analysis (LC-MS/MS) of a trypsin digest of the band appearing at approximately 60 kDa. Although only a preliminary investigation, this small study indicated the potential for this diagnostic approach [75]. 

### 5.7. Activity Assays

Assays for ribotoxins like ricin have been reported and were touched upon [71,72,73,74] in the mass spectrometry section above, when this method was used to probe the release of adenine by the *N*-glycosidase activity. Hale developed a microtitre plate based assay for the *N*-glycosidase activity expressed by ribotoxins based on the translation of luciferase mRNA. In samples containing active ribotoxin, luciferase mRNA was not translated and corresponding luminescence was decreased or absent [76]. Other approaches have reported measuring the enzymic activity using electrochemiluminescence [77], giving an assay which offered a similar level of sensitivity to that of an antibody based electrochemiluminescent assay (0.1 ng mL^−1^). An alternative method, that of *in vitro* assay, tests the full activity of ricin including the binding to cells, uptake and expression of toxicity. 

One example of this approach is the use of monkey kidney Vero cells in a toxicity assay. In this report [78] these cells were used in culture to assess the efficacy of monoclonal antibodies against ricin to protect the cells from toxicity, as assessed using MTT, an assay based on mitochondrial function. In an alternative approach [79], Vero cells in culture were used to assess the inhibition of protein synthesis by manipulated ricin molecules using the incorporation of [35S] methionine into cellular protein. There are opportunities to interrogate various aspects of cellular physiology or biochemistry following interaction with a toxic agent such as ricin, to probe the consequences of intoxication in detail. 

Defence against ricin toxin, therefore, involves an understanding of the hazards presented by the toxin when exposed by various routes and of the lesions resulting from exposure; this can help in the development of countermeasures against the toxin. If it is suspected that people may have been exposed to ricin, then it is essential to enable an early and robust diagnosis in order to start treating those who require appropriate therapeutic measures. To this end, we are testing the potential of immunoaffinity columns and immunochromatographic test strips, two quite rapid and sensitive assay systems, for supplying the necessary information. It is also important to be able to probe complex matrices such as foods in order to determine whether these have been contaminated with ricin. The best approach to ensure robust detection is to apply several orthogonal methods which are based on different criteria and this might include an antibody based test, a method based on the presence of specific nucleic acid (DNA or RNA) and a mass spectrometric analysis. It should be apparent that there is ample specific technology available to provide an unequivocal answer to this challenge. 
